# Use of Bioimpedance Monitoring and Artificial Intelligence in Heart Failure: A Scoping Review

**DOI:** 10.3390/bioengineering13101227

**Published:** 2026-10-20

**Authors:** Martin Puertas-Albarracín, Luis Giménez-Miranda, Javier Medrano, María del Carmen Romero-Ternero, Andreea M. Oprescu, Alberto Olmo

**Affiliations:** 1 Departamento de Tecnología Electrónica, Universidad de Sevilla, 41012 Sevilla, Spain; mpuertas-ibis@us.es (M.P.-A.); mcromerot@us.es (M.d.C.R.-T.); aolmo1@us.es (A.O.); 2 Instituto de Biomedicina de Sevilla (IBiS-US), 41013 Sevilla, Spain; luis.gimenez.sspa@juntadeandalucia.es (L.G.-M.); fmedrano@us.es (J.M.); 3 Hospital Universitario Virgen del Rocío (HUVR), 41013 Sevilla, Spain; 4 Consorcio de Investigación Biomédica en Red de Epidemiología y Salud Pública (CIBERESP), 41013 Sevilla, Spain; 5 Departamento de Medicina, Universidad de Sevilla, 41004 Sevilla, Spain; 6 Instituto de Ingeniería Informática (I3US), Universidad de Sevilla, 41012 Sevilla, Spain; 7 Instituto de Microelectrónica de Sevilla (IMSE-CNM-CSIC), 41092 Sevilla, Spain

**Keywords:** artificial intelligence, bioimpedance sensors, clinical decision support systems, heart failure, volume status, congestion, remote monitoring

## Abstract

Heart failure (HF) is frequently accompanied by congestion and fluid accumulation, which are key drivers of decompensation and hospitalization. Bioimpedance wearable sensors (BI), coupled with artificial intelligence (AI) and clinical decision-support systems (CDSS), offer an emerging opportunity to move HF volume-status assessment beyond conventional clinical monitoring towards earlier risk detection and personalized decision support. This scoping review mapped the available evidence on these integrated approaches. Following PRISMA-ScR guidance, systematic searches were conducted in Scopus, IEEE Xplore, PubMed, and Embase, with the final search performed on August 2, 2026. Ten studies were included, of which six applied AI techniques, two incorporated a CDSS, and two included both AI and CDSS. BI measurements were acquired using heterogeneous approaches, including implanted devices, wearables, and other non-invasive systems, and they encompassed intrathoracic impedance, thoracic BI, or whole-body bioimpedance analysis. AI applications focused on HF decompensation, fluid accumulation, pleural effusion, and the risks of hospitalization or readmission, whereas CDSS primarily provided risk information, alerts, or structured clinical decision support. Overall, the integration of BI sensors with AI or CDSS represents a rapidly developing transformative approach to HF volume-status management, with the potential to enable earlier intervention and more individualized clinical decision-making.

## 1. Introduction

Heart failure (HF) is frequently accompanied by disturbances in volume status, including congestion, fluid retention, and volume overload. These abnormalities may manifest as peripheral edema, pulmonary congestion, pleural effusion, or other forms of fluid accumulation and are commonly associated with HF decompensation and hospitalization [1,2]. Accurate assessment and early identification of changes in volume status are therefore important for timely intervention, treatment adjustment, and the prevention of adverse outcomes [3,4]. Renal dysfunction and cardiorenal interactions may further complicate fluid regulation in HF; however, the clinical relevance of volume-status assessment extends across the broader HF population.

Bioimpedance (BI) provides information on the electrical properties of biological tissues and has been investigated as a means of assessing body-water distribution and changes in thoracic or systemic fluid status [5]. Depending on the measurement configuration, BI can be obtained non-invasively using wearable devices or surface electrodes, or through implanted cardiac devices that measure intrathoracic impedance. In HF, BI-derived variables have been explored for assessment of congestion, body-water compartments, lung or thoracic fluid accumulation, and changes associated with clinical deterioration [1,6]. More recent work has also investigated the prognostic value of BI-derived total body water in acute decompensated HF, highlighting both its potential and the need for further clinical validation [7].

Artificial intelligence (AI), including machine learning (ML) and deep learning (DL), offers methods for identifying complex relationships in heterogeneous and longitudinal clinical data and for addressing tasks such as classification, prediction, regression, and risk stratification [8,9]. These characteristics are particularly relevant in HF, where physiological, clinical, laboratory, imaging, and device-derived information may change over time and jointly reflect evolving congestion or decompensation.

BI-derived signals and parameters can serve as inputs to AI models, either alone or in combination with other physiological and clinical variables. In HF, this integration has been explored for several clinically relevant purposes, including the detection of lung-fluid accumulation [10], the prediction of acute decompensation using remotely acquired transthoracic BI and ECG data [11], the estimation of pleural effusion using multi-frequency BI and machine learning [12], and the prediction of HF readmission using longitudinal implantable-device diagnostics, including intrathoracic impedance [13]. In parallel, BI-derived information has been incorporated into decision-support approaches ranging from probabilistic HF-hospitalization risk stratification [14] to impedance-triggered clinical management algorithms [15]. Together, these studies illustrate how BI can contribute to AI- and CDSS-supported assessment, monitoring, prediction, and management of HF-related volume status.

Recent evidence also highlights important limitations in the clinical application of BI. BI-derived measures may provide complementary information on congestion and fluid status beyond conventional clinical assessment. For example, BIA-derived total body water has been associated with subsequent cardiovascular events in patients with acute decompensated HF [7]. However, BI measurements can be influenced by measurement configuration, electrode placement, physiological conditions, and the monitoring context, and their clinical utility remains incompletely established. In wearable applications, technical reliability may also represent an important challenge; Reljin et al. reported a loss of usable recordings associated with technical and signal-quality limitations [10]. These limitations support the investigation of multiparametric and computational approaches capable of integrating BI with additional clinical and physiological information.

The role of these technologies also depends on how they are incorporated into clinical workflows. CDSS can provide patient-specific risk information, alerts, reminders, or proposed actions intended to assist healthcare professionals with assessment and management decisions [16]. In HF, decision-support approaches based on device-derived physiological measurements have been investigated for risk stratification, monitoring of clinical deterioration, and support for treatment decisions [14,15]. Broader initiatives such as STRATIFYHF further illustrate the growing interest in combining multimodal patient data, biomarkers, and advanced analytics within AI-driven decision-support frameworks for HF diagnosis, monitoring, and risk prediction [17].

Despite these developments, the available evidence remains limited and heterogeneous in terms of the clinical problem addressed, the BI configuration used, the variables incorporated into computational models, the AI task and architecture, the validation strategy, and the degree of clinical decision support provided. Moreover, many studies evaluate diagnostic or predictive performance rather than the effect of the technological intervention on subsequent hospitalization, mortality, or other adverse clinical outcomes. Consequently, the clinical role of combining BI with AI and/or CDSS in the assessment and management of HF-related volume status remains incompletely characterized.

The research question guiding this scoping review is: “What evidence exists on the combined use of bioimpedance and artificial intelligence or clinical decision support systems for the assessment, monitoring, prediction, and management of heart failure-related volume status?”

Accordingly, this review aims to map the available evidence across clinical and technological domains. From a clinical perspective, we examine the HF-related conditions and volume-status manifestations addressed, study populations, predictive variables, biomarkers, clinical outcomes, and adverse events targeted for prevention. From a technological perspective, we characterize the AI techniques, tasks, models, performance metrics, validation strategies, and CDSS characteristics reported in the included studies. We also examine BI measurement approaches, including measurement modality, sensing platform (implanted, wearable, or other non-invasive, non-wearable methods), monitoring configuration, electrode placement and characteristics, and measurement frequencies. By integrating these domains, this review provides a focused overview of how BI is currently combined with AI and/or CDSS for HF-related volume-status assessment, monitoring, prediction, and management.

The research question is addressed throughout the manuscript. Section 2 describes the methodological framework used to identify, select, and extract the relevant evidence. Section 3.1 characterizes the included evidence in terms of geographical distribution and publication trends. Section 3.2 presents the clinical perspective of HF-related volume status, including sample characteristics and the main conditions and adverse outcomes investigated. Section 3.3 summarizes the AI and CDSS approaches reported in the included studies, while Section 3.4 describes the bioimpedance measurements employed. Section 4 interprets the identified evidence, addressing the role of bioimpedance, wearable acquisition, AI applications, data sources and clinical integration, CDSS approaches, clinical impact, and current research gaps and challenges.

## 2. Materials and Methods

This scoping review was conducted in accordance with the Preferred Reporting Items for Systematic Reviews and Meta-Analyses extension for Scoping Reviews (PRISMA-ScR) [18].

### 2.1. Inclusion Criteria

Eligible studies were required to investigate the combined use of BI with AI techniques and/or a CDSS in relation to HF-related volume status. Applications of interest included the assessment, monitoring, prediction, management, or investigation of congestion, fluid accumulation, volume overload, or other manifestations of altered volume status in HF. The following inclusion criteria were applied: Studies published in English; Studies published between January 2006 and August 2026; Studies involving human participants or data associated with humans; Original research publications; and Studies using BI data together with AI techniques and/or a CDSS to assess, monitor, predict, manage, or investigate HF-related volume status.

### 2.2. Search Strategy and Research Databases

A broad search strategy was developed to maximize the retrieval of potentially relevant studies while remaining aligned with the research question, consistent with the scoping review approach described by [19]. Four databases were searched: Scopus, IEEE Xplore, PubMed, and Embase. No additional filters were applied at the database search stage. The same search strategy was used across Scopus, IEEE Xplore, PubMed, and Embase. The final search was conducted on 2 August 2026, using the following strategy:

(“artificial intelligence” OR “machine learning” OR “deep learning” OR “neural network*” OR “decision support” OR “expert system*” OR “intelligent system*” OR “fuzzy logic” OR “Bayesian network*”) AND (bioimpedance OR impedance) AND (“heart failure”). During the development of the search strategy, additional terms related to HF-associated fluid imbalance, volume overload, congestion, and fluid accumulation were considered. However, this conceptual block was not retained in the final search because requiring these terms could have excluded potentially relevant studies in which HF-related volume status was addressed without being explicitly described using this terminology. Relevance to HF-related volume status was therefore assessed during the screening according to the predefined eligibility criteria.

### 2.3. Screening Process

During the screening process, two reviewers independently evaluated the retrieved sources to determine their eligibility for inclusion. Titles, keywords, and abstracts were reviewed during the initial screening phase, and each source was classified as included or excluded according to the predefined eligibility criteria. Studies addressing topics such as malignant ventricular arrhythmia, dehydration, sarcopenia, visceral fat, or exercise-related water loss were excluded when they were not clearly related to HF or to the assessment, monitoring, prediction, or management of HF-related volume status.

Articles considered potentially eligible after title and abstract screening were subsequently assessed in full text to confirm eligibility. In cases of disagreement regarding the inclusion decision, a third reviewer was designated to resolve discrepancies. Once the final set of studies was selected, data extraction and charting were performed as described in the following subsection.

Inter-rater reliability between the two reviewers during the title and abstract screening phase was assessed using Cohen’s κ coefficient [20]. Inter-rater agreement was interpreted according to the Landis and Koch classification, where κ values are categorized as slight (0.00–0.20), fair (0.21–0.40), moderate (0.41–0.60), substantial (0.61–0.80), and almost perfect (0.81–1.00) [21].

The final search across the four databases yielded a total of 245 records. After removing 87 duplicate records, 158 unique records were screened. According to the predefined eligibility criteria, 81 records were excluded because they were reviews, book chapters, conference abstracts or collections, or other non-original publications. The remaining 77 original research records were assessed by title and abstract. Following this screening stage, 61 articles were excluded, and 16 articles proceeded to full-text eligibility assessment. During full-text assessment, the predefined reasons for exclusion were:

- Wrong or irrelevant topic: studies not aligned with the central theme of the review, particularly those not focused on HF-related volume status assessment, monitoring, prediction, or management.
- Use of inappropriate technology: studies using technologies outside the scope of BI-based assessment or monitoring, even when addressing edema, fluid accumulation, congestion, or HF-related conditions.
- Absence of AI or CDSS: studies that did not include implemented AI methods or decision support systems.
- Limited applicability to HF-related volume status: studies whose relevance to HF-related fluid accumulation, congestion, edema, volume overload, or fluid status was insufficient for inclusion.

**Figure 1:**
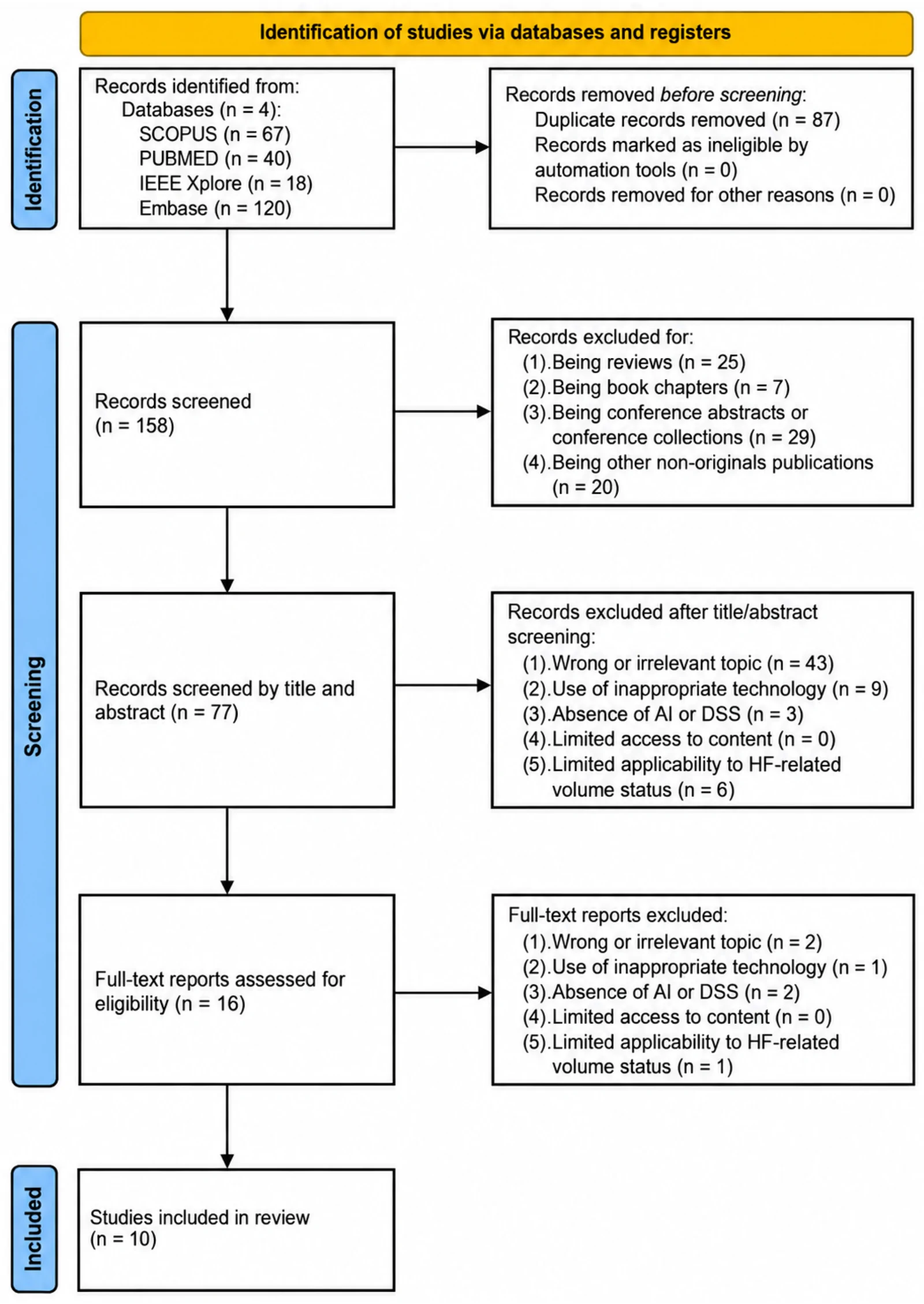
PRISMA flow diagram for this study: identification, screening, and final inclusion of studies.

Inter-rater reliability during the title and abstract screening phase was substantial, with a Cohen’s kappa of 0.77. A total of 6 of the 16 full-text reports were subsequently excluded, leaving 10 studies that fulfilled the eligibility criteria and were included in the scoping review. Figure 1 presents the PRISMA flow chart. A total of 10 articles were finally included in the review. The analysis is presented in the following sections.

### 2.4. Data Extraction

Study characteristics were first extracted, including the continent and country in which each study was conducted and the study period, when reported. To enable analysis across both clinical and technical domains, the remaining data items were organized into predefined categories. Clinical data included items related to population characteristics, monitoring and clinical assessment, outcomes and impact, and data collection. Technical data included items related to the methodological and technological characteristics of the systems and approaches evaluated.

To promote consistency during the data-extraction process, a standardized extraction form was developed in a shared Excel spreadsheet. The form consisted of predefined sections and fields derived from relevant literature and the research team’s expertise. The complete data-extraction chart is provided in the Supplementary Material. All three reviewers jointly reviewed the form and calibrated their interpretation of each field before full data extraction.

A pilot extraction was then conducted using two randomly selected articles. Each reviewer independently completed the extraction for these studies, and the resulting entries were compared. During this pilot phase, minor adjustments were made to improve the clarity and consistency of the extraction form, including: clarifying field definitions whose meaning was initially ambiguous, adding predefined response options to standardize categorical entries; separating items that combined multiple concepts; and reordering certain fields to improve the extraction workflow.

After these refinements, agreement was reached among the reviewers on the interpretation and application of the extraction fields. Each included study was subsequently extracted independently by two reviewers using rotating reviewer pairs. Extracted data were compared item by item, and discrepancies were resolved through discussion. When consensus could not be reached, a third reviewer adjudicated.

To ensure consistency and facilitate interpretation across readers from different disciplinary backgrounds, a simplified taxonomy of BI-related terminology is proposed in this review. Six categories were defined based on the measurement configuration, frequency spectrum, and clinical context in which the impedance signal is used:

- Bioelectrical Impedance Analysis (BIA): Conventional whole-body or segmental impedance measurement, typically using hand-to-foot or foot-to-foot electrodes. Commonly used to estimate body composition or fluid status.
- Thoracic BI (TH): Non-invasive impedance measured across the thorax with surface electrodes to monitor thoracic fluid content or respiratory dynamics.
- Intrathoracic impedance (ITH): Impedance measured by implanted cardiac devices (Implantable Cardioverter-Defibrillator (ICD)/Cardiac Resynchronization Therapy-Defibrillator (CRT-D)) through intracardiac leads and used as a device-derived indicator associated with changes in intrathoracic fluid status.
- Transthoracic bioimpedance (TTH): measuring changes in electrical resistance across the chest to evaluate cardiovascular function and fluid accumulation in the lungs.
- BI Spectroscopy (BIS): Multi-frequency impedance measurement over a wide frequency range, enabling estimation of extracellular and intracellular fluid compartments and improved tissue characterization.
- Unspecified electrical BI: Studies reporting “electrical BI” without details on electrode configuration, frequency domain, or measurement modality.

In addition, each study was classified according to the sensing platform used to acquire BI: implanted, when impedance was measured by an implanted device; wearable, when BI was acquired with a body-worn system; and non-invasive non-wearable, when surface electrodes were used without a body-worn system (such as electrodes mounted on measurement plates or whole-body BIA). Studies combining external and implanted acquisition were classified as implanted. Finally, to improve interpretability and reduce ambiguity regarding the proposed CDSS automation levels, the proposed automation-level classification was conceptually grounded in established human automation interaction models [22,23]. The six levels used in this review represent a simplified adaptation for clinical decision-support applications, ranging from no automation to fully autonomous action (see Table 1).

## 3. Results

### 3.1. Geographical Distribution and Publication Trend

**Figure 2:**
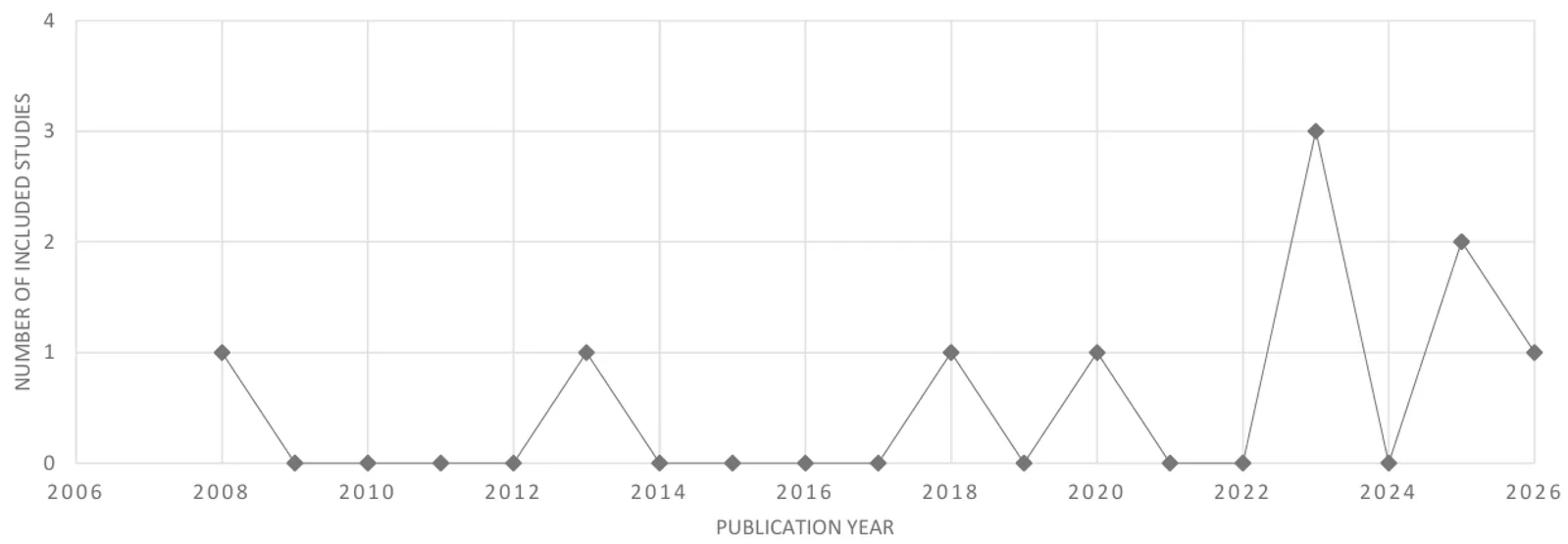
Temporal distribution of included studies by publication year. The search was conducted up to 2 August, 2026.

The 10 included studies were published between 2008 and 2026. One study was published in 2008 (*n* = 1, 10%) [15], one in 2013 (*n* = 1, 10%) [14], one in 2018 (*n* = 1, 10%) [27], and one in 2020 (*n* = 1, 10%) [10]. Two studies were published in 2025 (*n* = 2, 20%) [7,28], three in 2023 (*n* = 3, 30%) [11,12,29], and one in 2026 (*n* = 1, 10%) [13]. Overall, 6 of the 10 included studies were published from 2023 onward (*n* = 6, 60%). Figure 2 shows the temporal distribution of the included studies by publication year.

**Figure 3:**
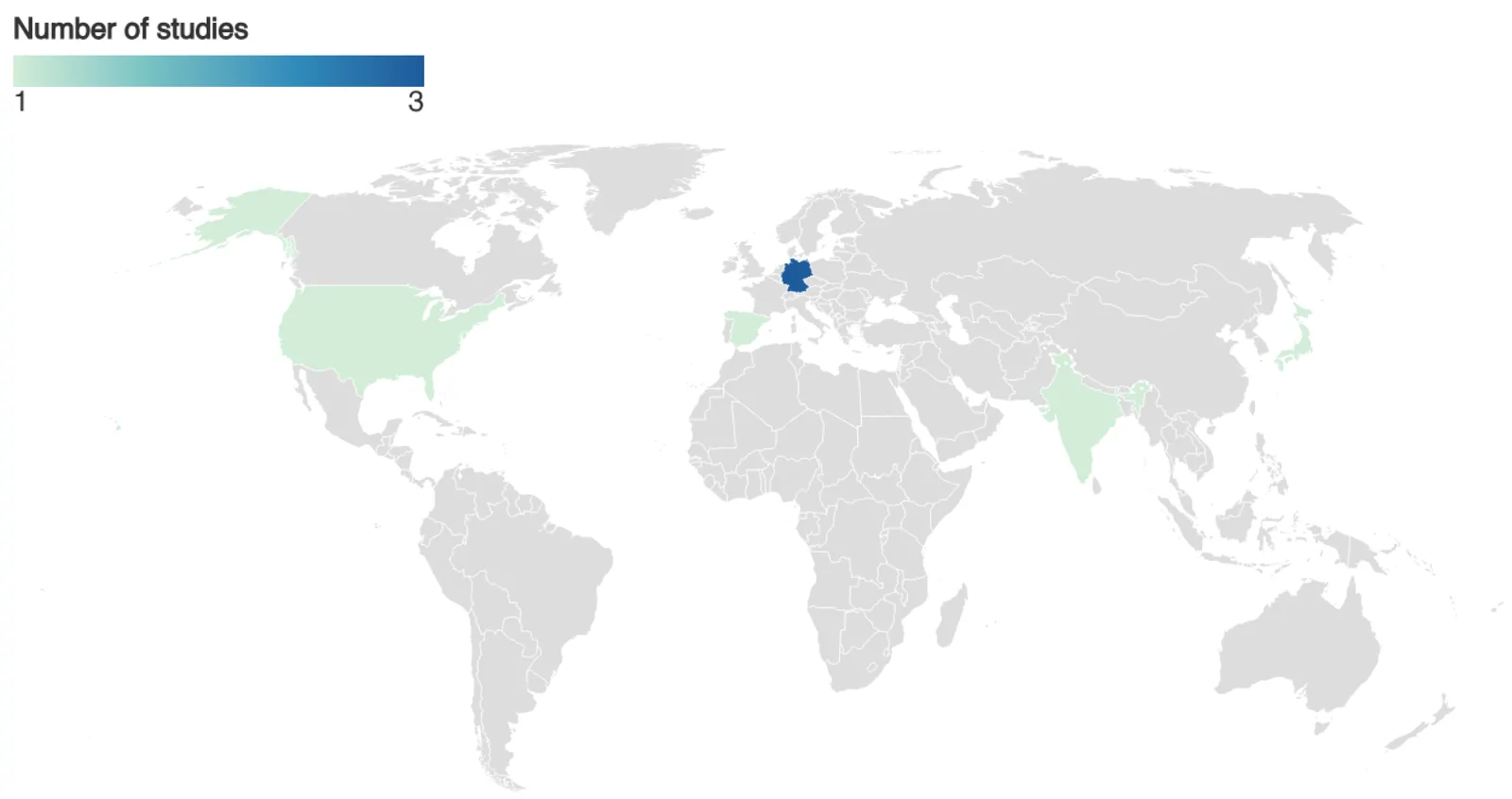
Geographical distribution of the included studies with explicitly reported study location.

Study location was explicitly reported in 6 of the 10 included studies (*n* = 6, 60%), whereas it was not clearly specified in four studies (*n* = 4, 40%). Three studies (*n* = 3, 30%) involved European populations: two were conducted in Germany [7,15], and one used data collected across six clinical centers in Germany and Spain [27]. One study (*n* = 1, 10%) was conducted in the United States [10], one (*n* = 1, 10%) in Japan [12], and one (*n* = 1, 10%) in India [28]. Study location was recorded as “Not specified” for the remaining four studies (*n* = 4, 40%) [11,13,14,29]. Figure 3 shows the geographical distribution of the included studies with explicitly reported study location.

### 3.2. Clinical Perspective of HF-Related Volume Status

#### 3.2.1. Sample Size and Characteristics

The included studies reported participant age using different approaches. In three studies (*n* = 3, 30%), age was reported primarily as an overall mean with standard deviation. Three studies (*n* = 3, 30%) reported age according to participant or analytical subgroups. Finally, two studies (20%) did not report participant age in the included article: Sarkar and Koehler [14] and Nunes et al. [27].

Sex reporting varied across the included studies. Quantitative sex distributions were reported in six of the 10 studies (*n* = 6, 60%), although the level of detail differed between studies. Three studies (*n* = 3, 30%) provided sex data stratified by clinical or analytical subgroups. Two studies (*n* = 2, 20%) reported sex distribution at the overall cohort level. Knackstedt et al. reported sex as absolute counts rather than percentages, with 26 male and 16 female participants among the 42 included patients [15]. These results are summarized in Table 2. Summary of the included studies with information on analysed sample or unit of analysis, age reporting, participant age, sex distribution, BI technique, and sensing platform.

#### 3.2.2. Main Conditions Studied and Targeted Adverse Outcomes for Prevention

The main conditions addressed across the included studies were related to HF and HF-related volume status, although the specific clinical context varied. Five studies (*n* = 5, 50%) investigated HF decompensation or acute decompensated HF [7,10,11,14,27]. Two studies (*n* = 2, 20%) focused on congestive or chronic HF in the context of congestion or fluid-status monitoring [15,29]. Two studies (*n* = 2, 20%) primarily addressed specific fluid-status manifestations, namely pleural effusion [12] and thoracic fluid accumulation [28]. Ramos et al. (*n* = 1, 10%) investigated the post-HF hospitalization setting, with subsequent HF readmission as the principal clinical event of interest [13]. Overall, the included evidence covered HF-related clinical contexts involving decompensation, congestion, fluid accumulation, and post-hospitalization HF events.

All 10 included studies addressed at least one adverse clinical outcome that the proposed monitoring, prediction, or decision-support approach was intended to detect early, anticipate, or potentially help prevent. Hospitalization or readmission was the most consistently identified adverse outcome, being relevant across all 10 studies (*n* = 10, 100%). Seven studies (*n* = 7, 70%) also addressed HF decompensation, worsening congestion, or other forms of fluid-related clinical deterioration [10,11,12,14,15,27,28]. Mortality was identified as a relevant adverse outcome in three studies (*n* = 3, 30%) [7,11,29]. Pan et al. additionally considered emergency-department visits among the adverse clinical events associated with recurrent ADHF [11].

**Figure 4:**
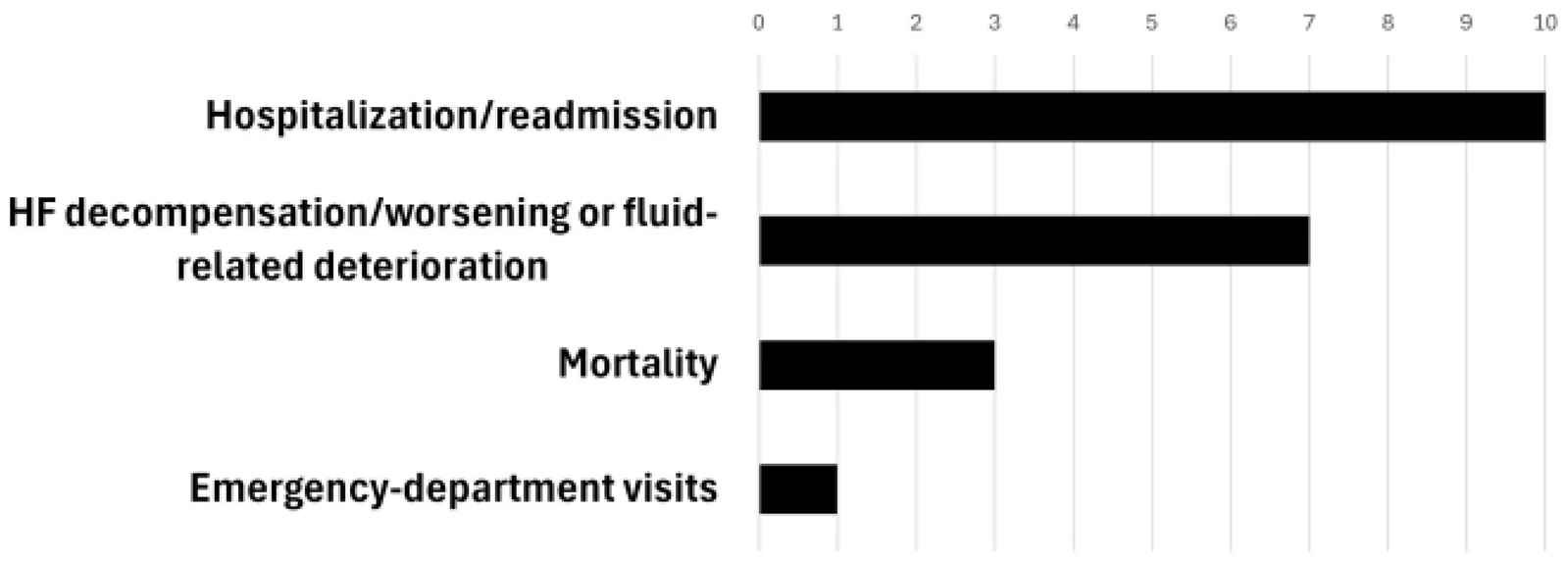
Adverse clinical outcomes targeted for prevention in the included studies.

Because individual studies could target more than one adverse outcome, these categories were not mutually exclusive. Furthermore, in several studies these outcomes represented intended clinical targets or motivations for early detection rather than outcomes for which a preventive effect of the technological intervention was directly evaluated. The distribution of the adverse outcomes targeted for prevention is summarized in Figure 4.

### 3.3. AI and CDSS in HF-Related Volume Status Assessment and Management

Of the 10 included studies, eight implemented an AI-based method (*n* = 8, 80%), whereas four incorporated a CDSS or structured clinical decision-support component (*n* = 4, 40%). Two studies included both AI and CDSS components (*n* = 2, 20%): Sarkar and Koehler and Nose et al. [12,14]. Knackstedt et al. and Zile et al. incorporated decision-support approaches without implementing a new AI model in the included study [15,29]. The analysis of AI-related characteristics therefore focused on the eight studies that implemented AI methods. Their main characteristics are summarized in Table 3.

Among the eight studies implementing AI, binary classification was the most frequent task (*n* = 5, 62.5%), being used by Nunes et al., Reljin et al., Pan et al., Nose et al., and Ramos et al. [10,11,12,13,27]. Regression was used in two studies (*n* = 2, 25%), although for different purposes: Pan et al. used an MLP regressor as part of its cross-modal feature-learning pipeline, whereas Yadav et al. used a graph-based neural network to estimate thoracic fluid percentage [11,28]. Reljin et al. additionally evaluated multiclass classification (*n* = 1, 12.5%) to discriminate among control, baseline, and discharge conditions [10]. Sarkar and Koehler used a Bayesian belief network for probabilistic HF-hospitalization risk stratification (*n* = 1, 12.5%) [14], whereas Faragli et al. used neural-network analysis for outcome prediction and assessment of predictor importance for a composite cardiovascular endpoint (*n* = 1, 12.5%) [7]. Because individual studies could address more than one AI task, these categories were not mutually exclusive.

The AI methods were methodologically heterogeneous. Neural-network-based approaches were implemented in four studies (*n* = 4, 50%), although with different architectures and purposes. Pan et al. used pretrained DenseNet121 and VGG19 architectures together with an MLP regressor and SVM classifier [11]; Faragli et al. applied radial-basis and multilayer perceptron neural networks [7]; Yadav et al. developed a graph neural network incorporating graph-attention mechanisms [28]; and Ramos et al. compared 1D-CNN and LSTM architectures [13]. SVM-based classification was used in two studies (*n* = 2, 25%) [10,11]. The remaining approaches included a Fisher discriminant classifier combined with genetic-algorithm feature selection (*n* = 1, 12.5%) [27], LightGBM (*n* = 1, 12.5%) [12], and a Bayesian belief network (*n* = 1, 12.5%) [14]. Reljin et al. additionally evaluated KNN and decision-tree classifiers [10]. Since several studies evaluated or combined more than one model, model frequencies were not mutually exclusive.

Validation strategies also varied substantially. Some form of cross-validation was used in five of the eight AI studies (*n* = 5, 62.5%) [10,11,12,13,27]. Nunes et al. used Leave-One-Out validation, whereas Reljin et al. used Leave-One-Subject-Out cross-validation [10,27]. Pan et al. used 5-fold cross-validation, Nose et al. used 4-fold cross-validation with subject-level separation, and Ramos et al. used stratified 10-fold cross-validation for hyperparameter optimization [11,12,13]. Ramos et al. additionally evaluated the final models on an independent held-out test partition with no patient overlap between the training, validation, and testing subsets [13]. Yadav et al. used separate training, validation, and test sets (*n* = 1, 12.5%), although external validation was not reported [28]. Sarkar and Koehler developed and evaluated the Bayesian model using the same source dataset (*n* = 1, 12.5%) and explicitly stated that independent validation was still required [14]. The validation procedure was not clearly specified for the neural-network analysis in Faragli et al. (*n* = 1, 12.5%) [7]. Overall, validation was predominantly internal; no study clearly reported external validation of the AI model in an independent external cohort.

Performance reporting was heterogeneous and reflected the different AI tasks. Accuracy was reported in four studies (*n* = 4, 50%) [10,11,12,13]. Sensitivity and specificity were each reported in four studies (*n* = 4, 50%) [12,13,14,27]. AUC-based measures were reported in three studies (*n* = 3, 37.5%): AUC-PR in Pan et al. and ROC-AUC/AUC in Nose et al. and Ramos et al. [11,12,13]. Nunes et al. additionally reported Gmean (*n* = 1, 12.5%), while Pan et al. reported precision, recall, and F1-score (*n* = 1, 12.5% for each metric) [11,27]. Yadav et al., whose task involved continuous thoracic-fluid estimation, reported MSE, MAE, and $R^{2}$ (*n* = 1, 12.5% for each metric) rather than classification metrics [28]. Faragli et al. primarily reported normalized predictor importance from the neural-network analysis rather than conventional discrimination metrics [7]. Because individual studies frequently reported multiple performance measures, metric frequencies were not mutually exclusive.

The AI models relied predominantly on physiological and bioimpedance data obtained from monitoring or measurement devices, which were used in all eight AI studies (*n* = 8, 100%). These included implantable-device diagnostics, transthoracic or intrathoracic bioimpedance, ECG and heart-rate variability signals, whole-body BIA measurements, and wearable multifrequency bioimpedance data. Additional data sources were incorporated in a smaller number of studies. Clinical or demographic information was used together with device-derived data in some models, particularly in Nose et al., Faragli et al., and Yadav et al. [7,12,28]. Laboratory variables were incorporated in Nose et al. and Faragli et al. [7,12], while imaging-derived information was incorporated in the study framework of Faragli et al. and Yadav et al. [7,28]. Ramos et al. combined longitudinal CIED data with linked electronic health record information for identification of HF hospitalization and readmission outcomes, although the predictive model inputs themselves consisted of device-derived time-series diagnostics [13].

Of the 10 included studies, four incorporated a CDSS or structured clinical decision-support component (*n* = 4, 40%) [12,14,15,29]. Two of these studies also implemented AI methods (*n* = 2, 20% of all included studies; 50% of studies with CDSS): Sarkar and Koehler and Nose et al. [12,14]. The identified systems differed in their clinical purpose, intervention type, data sources, intended users, and degree of automation. Their main characteristics are summarized in Table 4.

Overall, three of the four CDSS or decision-support components were classified as automation Level 1 (*n* = 3, 75%) [12,14,29], whereas no included system was classified as Level 4 or Level 5. Thus, none of the identified approaches autonomously executed therapeutic decisions, and clinician interpretation or confirmation remained necessary when clinical action was required.

To explore the potential implementation implications of the CDSS and decision-support components identified in this review, their reported characteristics were mapped to relevant domains of the Consolidated Framework for Implementation Research (CFIR) [30]. This synthesis should be interpreted as a descriptive mapping rather than as a formal CFIR-based implementation evaluation, as the included studies were not specifically designed to assess implementation determinants. Recurrent implementation considerations included limited or unreported integration with EHR, predominantly low levels of automation, dependence on device-specific physiological data, and incompletely specified end-user roles. Potential facilitators included the use of objective bioimpedance-derived measurements, multimodal physiological information, remote or cloud-based infrastructures, and patient-specific risk information. These findings are summarized in Table 5.

### 3.4. BI Measurements and Sensing Platforms in HF-Related Volume Status Assessment and Management

Bioimpedance was used in all 10 included studies (*n* = 10, 100%), although the measurement configuration and anatomical location varied considerably. Intrathoracic or internal thoracic impedance measurements were reported in five studies (*n* = 5, 50%) [13,14,15,27,29]. Thoracic or transthoracic BI was also used in five studies (*n* = 5, 50%) [10,11,12,27,28]. Faragli et al. used whole-body BIA/BIVA for assessment of body-water compartments (*n* = 1, 10%) [7]. These categories were not mutually exclusive because Nunes et al. included both external and internal thoracic impedance measurements.

The implanted approaches measured impedance through cardiac-device configurations. Knackstedt et al. measured intrathoracic impedance between the right-ventricular defibrillation coil and the implanted CRT-D generator [15]. Sarkar and Koehler described a measurement across the thorax between an intracardiac electrode and the implanted device [14]. Zile et al. and Ramos et al. similarly used right-ventricular lead-to-device-can measurements [13,29]. Nunes et al. incorporated both external and internal thoracic impedance as part of the multiparametric telemonitoring dataset, although the specific electrode configuration was not reported [27].

Among the non-implanted approaches, the number of electrodes was explicitly reported in four studies (*n* = 4, 40%). Reljin et al., Pan et al., and Nose et al. each used four-electrode configurations (*n* = 3, 30%) [10,11,12], whereas Yadav et al. used a 10-electrode thoracic belt (*n* = 1, 10%) [28]. The remaining six studies (*n* = 6, 60%) did not report an explicit electrode count. In implanted-device studies, the articles generally described the electrical measurement pathway between the cardiac lead and device can rather than reporting a conventional surface-electrode count.

The electrical measurement frequency was explicitly reported in four studies (*n* = 4, 40%). Reljin et al. performed multi-frequency transthoracic measurements at 16 frequencies ranging from 10 to 999 kHz [10]. Nose et al. measured resistance and reactance at 33 frequencies ranging from 2.5 to 350 kHz [12]. Yadav et al. acquired thoracic BI measurements at 25, 50, 75, 100, 125, and 150 kHz [28]. Faragli et al. used a single-frequency whole-body BIA measurement at 50 kHz with an applied current of 800 µA [7].

Regarding the sensing platform used to acquire BI measurements, five studies relied on implanted, and therefore invasive, intrathoracic impedance measurements (*n* = 5, 50%) [13,14,15,27,29], including Nunes et al., who combined external and internal thoracic impedance [27]. The remaining five studies used non-invasive acquisition systems (*n* = 5, 50%): three wearable systems, namely thoracic vests reported by Reljin et al. and Pan et al. [10,11] and a modular thoracic belt reported by Yadav et al. [28]; Lastly, two non-invasive non-wearable configurations, namely plate-mounted electrodes [12] and a whole-body BIA configuration [7].

### 3.5. Active Patient Participation

Active patient participation in data collection was identified in seven of the 10 included studies (*n* = 7, 70%) [7,10,11,12,15,27,28]. The nature of participation varied according to the monitoring strategy. In Knackstedt et al., patients were expected to recognize the acoustic device alert and contact or attend the CRT clinic for subsequent assessment [15]. In Nunes et al., patients were requested to perform daily physiological measurements as part of ambulatory tele-monitoring [27]. Reljin et al. required participants to wear the transthoracic BI vest during standardized recording sessions [10], while participants in the parent study analyzed by Pan et al. regularly used a wearable vest for post-discharge measurements [11]. Nose et al., Faragli et al., and Yadav et al. also required participants to undergo repeated or protocol-defined BI measurements [7,12,28].

Active participation was not required in three studies (*n* = 3, 30%) [13,14,29]. Sarkar and Koehler relied on diagnostic information collected and transmitted by implanted devices without requiring active patient compliance for the measurements [14], whereas Zile et al. and Ramos et al. retrospectively analyzed routinely collected device and remote-monitoring data [13,29]. Overall, patient participation requirements depended strongly on the BI configuration: external wearable and on-site measurement approaches generally required direct participant involvement, whereas implanted-device monitoring allowed physiological data to be collected with little or no active patient input.

## 4. Discussion

This scoping review identified 10 studies investigating the combined use of BI with AI and/or CDSS for the assessment, monitoring, prediction, or management of HF-related volume status. The available evidence was heterogeneous in terms of BI modality, clinical application, analytical approach, and validation strategy. Although the included studies illustrate several potential applications of BI-derived information for detecting fluid-related changes and supporting HF risk assessment, the limited evidence base and methodological heterogeneity constrain conclusions regarding the generalizability and clinical effectiveness of these approaches.

### 4.1. Role of BI in HF Applications

BI provided physiological information related to fluid status across the included HF applications, although the specific measurements and their role within the analytical models varied substantially. BI-derived information was used alone or in combination with other physiological, clinical, laboratory, or device-derived variables [7,10,11,12,13,14,27,28]. This heterogeneity reflects a predominantly multimodal approach rather than reliance on a single BI-derived parameter.

Thoracic and intrathoracic BI configurations predominated across the included studies [10,11,12,13,14,15,27,28,29], whereas whole-body BIA was represented by Faragli et al. [7]. The technologies nevertheless differed substantially, ranging from implanted-device impedance monitoring to wearable thoracic vests and belts, and whole-body BIA. This heterogeneity limits direct comparison of BI-derived variables across studies.

Electrode characteristics also varied between the non-invasive systems. Reljin et al. used reusable copper-mesh carbon black/polydimethylsiloxane (PDMS) dry electrodes incorporated into a wearable thoracic vest [10]. Pan et al. used textile electrodes integrated into a wearable vest [11], whereas Nose et al. used single-use ECG electrodes mounted on the measurement plates [12]. Yadav et al. developed a modular wearable belt containing sliding electrode modules that could be adjusted according to chest size [28]. The specific electrode material was not clearly reported by Nunes et al. or Faragli et al. In the implanted-device studies, electrode characteristics were primarily described in terms of the intracardiac lead/device-can measurement configuration rather than surface-electrode materials.

**Figure 5:**
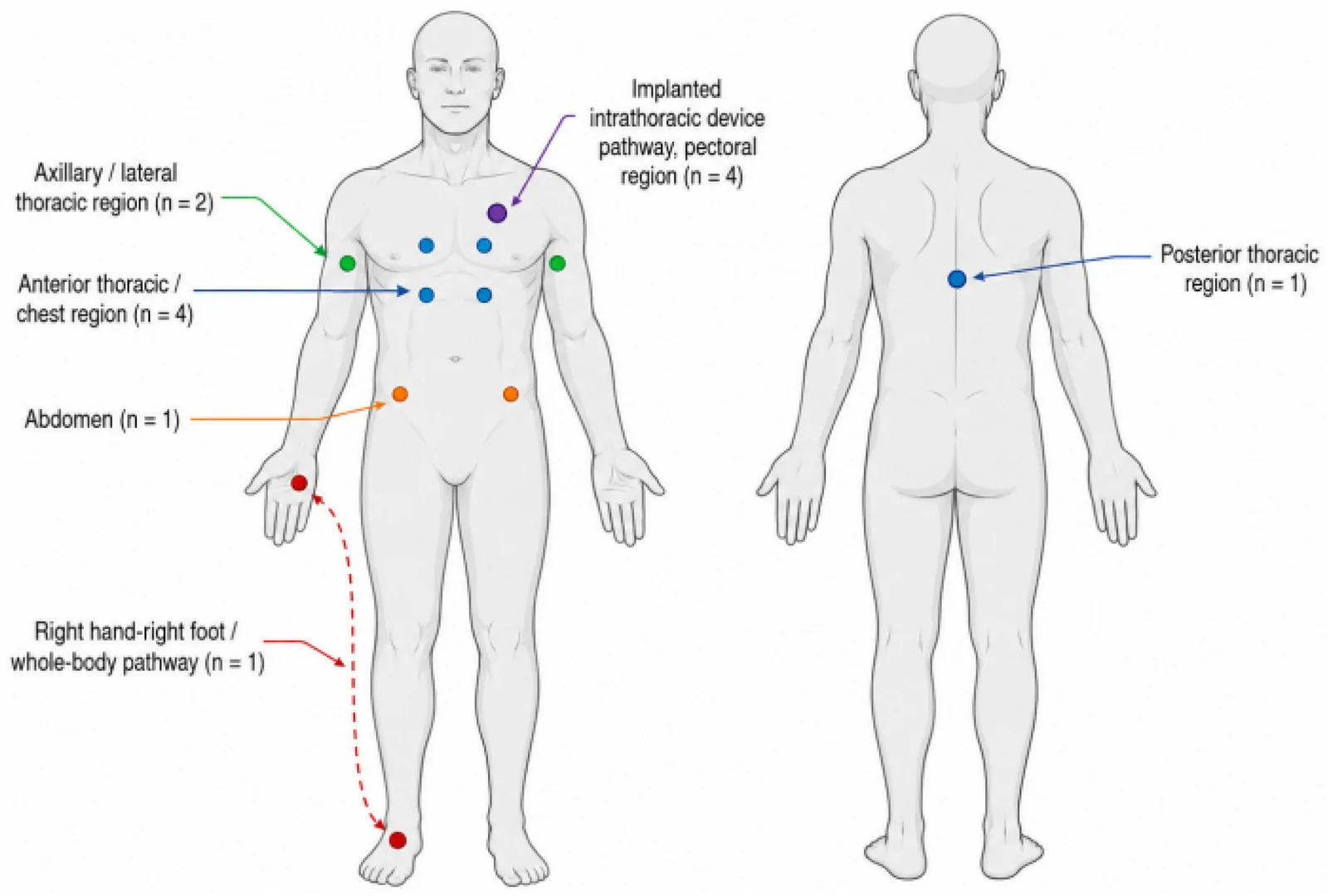
Electrode positions and impedance-measurement pathways identified in the included studies. The *n* values indicate the number of studies associated with each anatomical region or measurement pathway. Counts are not mutually exclusive because individual studies may contribute to more than one region.

Electrode placement or the corresponding impedance-measurement pathway was reported in eight studies (*n* = 8, 80%). Abdominal electrode placement was reported by Reljin et al. [10], whereas bilateral axillary placement was reported by Nose et al. [12]. Faragli et al. used a right hand-to-foot whole-body BIA configuration [7], and Yadav et al. used a thoracic belt spanning anterior, posterior, and lateral thoracic regions [28]. Pan et al. used a thoracic wearable vest with four textile electrodes, while Nunes et al. reported external thoracic impedance; in both cases, the precise anatomical position of each electrode was not specified [11,27]. Implanted approaches used intracardiac lead-to-device configurations [13,14,15,29]. These anatomical regions and measurement pathways are summarized in Figure 5.

Considerable variability was also observed in electrode placement, measurement pathways, operating frequencies, and acquisition protocols. External systems used different anatomical configurations, including thoracic, axillary, abdominal, and hand-to-foot measurements [7,10,11,12,28], whereas implanted systems relied on intracardiac-to-device impedance pathways [13,14,15,29]. In addition, the precise anatomical position of the electrodes was not consistently reported across studies. Such methodological variation may affect the comparability of BI measurements and represents an important challenge for the development and generalizability of AI models across devices, populations, and clinical settings. More standardized acquisition and reporting of BI measurements would facilitate reproducibility and comparability across future studies.

### 4.2. Wearable BI Acquisition

The three studies that reported wearable systems shared a distinctive profile [10,11,28]. They were among the most recent contributions, published from 2020 onward. All three implemented AI methods and reported the number of electrodes used, and two of them also reported the measurement frequency [10,28]. They were evaluated in the smallest cohorts of the review, between 8 and 60 participants, whereas the largest datasets all derived from implanted devices [13,14,29] and none of them incorporated a decision-support component, which was present only in implanted-device studies [14,15,29] and in one non-wearable non-invasive system [12]. Within this small set of studies, wearable acquisition was associated with more recent publication dates, more detailed reporting of acquisition parameters, and the integration of cardiac electrical signals, but also with smaller cohorts and the absence of decision-support components.

### 4.3. Application of AI in HF Studies

Among the eight studies implementing AI, binary classification was the most frequent analytical task [10,11,12,13,27], although regression [11,28], multiclass classification [10], probabilistic risk stratification [14], and outcome prediction [7] were also represented. The models were similarly heterogeneous, including neural-network-based architectures, SVMs, a Bayesian belief network, LightGBM, a Fisher discriminant classifier with genetic-algorithm-based feature selection, and graph-based approaches. This diversity reflects the different clinical objectives addressed, ranging from detection of fluid accumulation and HF decompensation to thoracic-fluid estimation and prediction of hospitalization or readmission risk.

No single modelling approach clearly emerged as dominant or superior. Differences in study populations, BI acquisition methods, sample sizes, prediction targets, and validation procedures preclude meaningful direct comparisons between algorithms. Consequently, the available evidence is better interpreted as demonstrating methodological exploration of different AI approaches rather than establishing an optimal modelling strategy for BI-based HF assessment.

Beyond predictive performance, interpretability remains relevant to the clinical translation of AI-based decision support. Complex models may provide limited insight into the factors underlying individual predictions, which can hinder their interpretation by healthcare professionals [31]. Future studies should therefore consider not only model discrimination and prediction performance but also how model outputs can be interpreted and incorporated into clinical decision-making.

Validation strategies varied substantially across the AI studies. Cross-validation was the most frequently used approach [10,11,12,13,27], although the specific procedures differed, including Leave-One-Out, Leave-One-Subject-Out, and k-fold strategies. Yadav et al. used separate training, validation, and test evaluations [28], whereas Sarkar and Koehler developed and evaluated their model using the same source dataset [14]. Ramos et al. additionally assessed the final models in a held-out test partition with no patient overlap between the training, validation, and testing subsets [13]. Despite these efforts to assess model performance, no study clearly reported external validation of an AI model in an independent external cohort.

This predominance of internal validation represents an important limitation for clinical translation. Performance obtained within the development population may not be maintained when models are applied to patients from different institutions, devices, acquisition protocols, or clinical settings. Independent external validation should therefore be prioritized in future studies, particularly given the substantial heterogeneity observed in BI acquisition and study populations.

Performance reporting was heterogeneous across the AI studies and reflected the diversity of analytical tasks. Accuracy, sensitivity, and specificity were among the most frequently reported classification metrics, whereas AUC-based measures were reported less consistently [10,11,12,13,14,27]. Other measures, including precision, recall, F1-score, and Gmean, were reported only in individual studies. For the continuous thoracic-fluid estimation task, Yadav et al. reported MSE, MAE, and $R^{2}$ [28], while Faragli et al. primarily reported predictor importance rather than conventional discrimination metrics [7].

This variability limits direct comparison of model performance across studies, particularly because the models addressed different clinical tasks and were evaluated using different validation strategies. Accuracy alone may also provide an incomplete representation of clinical performance, especially in imbalanced classification problems, where sensitivity, specificity, predictive values, and measures of discrimination may provide more informative complementary information. Future studies would benefit from more consistent and task-appropriate performance reporting, together with measures that facilitate interpretation of model performance in clinically relevant terms.

### 4.4. Data Sources and Clinical Integration

The AI studies relied predominantly on physiological and BI-derived information obtained from monitoring or measurement devices. These data included intrathoracic or transthoracic impedance, implantable-device diagnostics, ECG and heart-rate variability signals, whole-body BIA, and wearable multi-frequency BI measurements. Additional clinical, demographic, laboratory, or imaging information was incorporated in a smaller number of studies [7,12,28]. Ramos et al. additionally linked longitudinal CIED data with EHR information to identify HF hospitalization and readmission outcomes, although the predictive model inputs themselves were based on device-derived time-series diagnostics [13].

The predominance of device-derived physiological data is consistent with the monitoring-oriented nature of the included applications, but it also highlights challenges for clinical implementation. Differences in devices, acquisition procedures, data formats, and measurement environments may complicate interoperability and the transferability of models across settings. The integration of BI-derived measurements with routinely available clinical information may provide a more comprehensive representation of HF-related volume status, although the added value of individual data modalities requires further evaluation.

Future work should therefore prioritize standardized BI acquisition and reporting, improved interoperability between monitoring technologies and clinical information systems, and prospective evaluation of these approaches within routine clinical workflows.

### 4.5. CDSS Approaches for HF-Related Volume Status Management

Only four of the included studies incorporated a CDSS or structured decision-support component [12,14,15,29]. Their functions were limited in terms of automation: three were classified as Level 1, providing information or suggestions to support clinical interpretation, whereas one was classified as Level~2 because it incorporated an automated alert [15]. None of the identified systems reached Levels 3–5, indicating that BI-based decision support in this field remains focused on informing or alerting clinicians rather than automatically proposing or implementing therapeutic actions.

The CDSS evaluated by Knackstedt et al. was designed to integrate automatic intrathoracic impedance monitoring into clinical decision-making for patients with CHF [15]. An implanted CRT-D generated an acoustic alert when the OptiVol fluid index exceeded a predefined threshold. Following an alert, a staged clinical algorithm incorporated intrathoracic impedance, clinical evaluation, and BNP measurements to classify patients and propose specific management actions. Sarkar and Koehler developed a Bayesian belief network that combined implantable-device diagnostics into a probabilistic risk score for HF hospitalization within the following 30 days [14]. The model incorporated intrathoracic impedance, atrial fibrillation burden, ventricular rate during atrial fibrillation, night-time heart rate, heart-rate variability, and patient activity. The resulting risk score was intended to support clinician triage by identifying patients requiring greater or lesser clinical attention. Because the system provided patient-specific risk information without automatically recommending or executing a specific clinical action, it was classified as automation Level 1. The system operated independently of an EHR, and the intended users were clinicians. Nose et al. developed a machine-learning-based web system for estimating pleural effusion using multifrequency transthoracic bioimpedance together with clinical and laboratory variables [12]. The system consisted of a data-input component, cloud-based processing, and an information-display interface. As the system provided diagnostic or estimation information without automatically proposing or executing a therapeutic intervention, it was classified as automation Level 1. The system operated independently of an EHR. Zile et al. investigated the temporal characteristics of the TriageHF integrated risk metric and its individual device-derived components in patients with chronic HF [29]. The TriageHF score combined multiple device-derived variables, including intrathoracic impedance, nighttime heart rate, activity, heart-rate variability, atrial fibrillation burden, ventricular rate during atrial fibrillation, CRT pacing, and ventricular tachycardia episodes and shocks, into an integrated risk estimate. In the included study, these risk metrics were evaluated for HF-event risk stratification and characterization of recovery before, during, and after HF hospitalization rather than for autonomous modification of treatment. The system was therefore classified as automation Level 1. EHR integration and the specific intended end-user were not clearly specified.

This limited degree of automation is consistent with the need for clinical interpretation of HF-related congestion and changes in volume status, which may require consideration of symptoms, laboratory findings, treatment history, and other contextual information beyond BI measurements alone. Accordingly, the absence of higher levels of automation should not necessarily be interpreted as a technological deficiency, but rather as evidence that current systems remain primarily clinician-facing decision-support tools. The small number of CDSS studies also indicates that this area is less developed than the use of AI for prediction or classification.

### 4.6. Clinical Impact

The included studies suggest that combining BI with AI or CDSS may support several clinically relevant functions, including detection of fluid accumulation, identification of HF decompensation, estimation of thoracic fluid, and stratification of hospitalization or readmission risk [7,10,12,13,14,27,28]. These applications are clinically relevant because changes in fluid status and congestion may precede overt deterioration and could potentially provide an opportunity for earlier assessment or intervention.

However, evidence of direct clinical benefit remains limited. Most included studies evaluated diagnostic, classification, estimation, or predictive performance rather than whether use of the proposed technology reduced HF hospitalization, readmission, mortality, or other adverse outcomes. Knackstedt et al. incorporated intrathoracic impedance alerts into clinical management and evaluated subsequent hospitalization patterns, but the observed reduction in hospitalization was not statistically significant [15]. Consequently, the available evidence supports the potential clinical utility of BI combined with AI or CDSS but does not yet establish that these approaches improve patient outcomes.

This distinction between predictive performance and clinical effectiveness is particularly important for future research. Prospective studies should evaluate not only whether models accurately identify patients at risk, but also whether acting on the information they provide changes clinical decisions, enables timely treatment adjustment, reduces adverse events, or improves patient-centered outcomes. Such evaluations will be necessary to determine the added value of these technologies beyond conventional HF monitoring and risk assessment.

### 4.7. Research Gaps and Current Challenges

The limitations reported across the included studies reflected several recurring methodological concerns, particularly limited sample sizes, restricted generalizability, and incomplete validation. These issues were especially relevant to the AI studies, in which differences in population size, data availability, and analytical design limited the extent to which model performance could be generalized beyond the development setting.

Several challenges and research gaps emerge from the available evidence. First, BI acquisition was highly heterogeneous across the included studies, with differences in measurement modality, electrode configuration, anatomical location, operating frequency, and monitoring strategy. This variability complicates direct comparisons between studies and may limit the transferability of AI models across devices and clinical settings. More standardized acquisition and reporting of BI measurements would facilitate reproducibility and comparison across future studies.

Second, the available AI evidence was predominantly based on internal validation, with no clearly reported independent external validation. Together with differences in study populations and analytical datasets, this limits conclusions regarding model generalizability and represents an important barrier to clinical translation.

Clinical integration also remains insufficiently evaluated. The included studies primarily focused on measurement, detection, classification, risk estimation, or monitoring performance, whereas evidence regarding integration into routine clinical workflows and its subsequent effects on clinical decision-making or patient outcomes was limited. Similarly, although remote and wearable monitoring approaches were represented, the feasibility of their sustained use across broader HF populations requires further prospective evaluation.

These findings suggest that future research should prioritize standardized BI acquisition and reporting, independent validation in diverse populations and settings, clearer descriptions of device and patient interaction requirements, and prospective assessment of how BI-based AI or CDSS approaches function within real clinical workflows.

## 5. Conclusions

The combined use of bioimpedance with artificial intelligence and/or clinical decision support systems represents an emerging but still limited and heterogeneous evidence base in HF-related volume-status assessment. Studies have explored a broad range of BI modalities, including implanted intrathoracic impedance, non-invasive thoracic BI, and whole-body bioimpedance analysis, across applications ranging from fluid-status assessment and detection of HF decompensation to risk stratification and prediction of hospitalization or readmission. AI-based approaches were more frequently represented than CDSS, while the identified decision-support systems operated at relatively low levels of automation, primarily providing information, recommendations, or alerts rather than automated therapeutic actions. AI models also showed substantial heterogeneity in analytical tasks, architectures, performance metrics, and validation strategies. Importantly, no AI study clearly reported independent external validation in a separate cohort, highlighting a major gap between model development and clinical readiness.

Despite demonstrating the feasibility and potential clinical utility of integrating BI-derived information with AI or CDSS, the current evidence remains insufficient to establish an impact on patient outcomes. Most studies focused on detection, estimation, classification, or predictive performance rather than demonstrating reductions in hospitalization, readmission, mortality, or other adverse clinical outcomes. Thus, while these technologies hold considerable promise for enabling earlier identification of volume-related deterioration and more individualized HF monitoring, their clinical benefit remains to be established.

The next step for the field is therefore not simply to develop more sophisticated models, but to demonstrate that they improve clinical decision-making and patient outcomes. Future research should prioritize standardized BI acquisition and reporting, larger and more diverse populations, independent external validation, and prospective evaluation within real-world clinical workflows. Establishing whether BI-enabled AI and CDSS can provide clinically meaningful benefits beyond conventional HF monitoring will be critical to translating their technological promise into routine clinical practice.

## Figures and Tables

**Table 1:** Proposed description of CDSS automation levels, elaborated based on [22,23,24,25,26].

Level	Decision	Description
Level 0	No automation	No automated support is provided.
Level 1	Suggestions	Provides diagnostic or treatment suggestions; final decision is made by the clinician.
Level 2	Alerts/reminders	Automatically issues alerts or reminders; requires clinician action.
Level 3	Action proposals	Proposes specific actions that must be reviewed and confirmed by the clinician.
Level 4	Semi-autonomous	Executes actions automatically, while allowing clinician oversight and intervention.
Level 5	Fully autonomous	Executes actions independently without human intervention.

**Table 2:** Summary of the included studies with information on analysed sample or unit of analysis, age reporting, participant age, sex distribution, BI technique, and sensing platform.

Sample/Unit of Analysis	Reporting Method	Patient Age	Sex Percentage	BI Technique	Sensing Platform	Ref.
49	Mean with standard deviation	73 ± 11 years	84% men, 16% women	BIA	Non-invasive, non-wearable	[7]
60	Mean with standard deviation by condition	Control: 71.5 ± 8.5 years; baseline ADHF: 72.0 ± 10.7 years; discharge ADHF: 72.4 ± 9.0 years	Control: 59% men, 41% women; acute decompensated HF: 64% men, 36% women	TTH	Wearable	[10]
8 participants (441 ECG samples in final classifier dataset)	Inclusion criterion	≥21 years; descriptive age statistics for the analyzed participants were NS	NS	TTH	Wearable	[11]
145	Mean with standard deviation by analytical condition	Pleural effusion+: 86.3 ± 9.1 years; pleural effusion-: 63.9 ± 23.0 years; healthy-control datasets: 44.8 ± 13.2 years; discharge without pleural effusion: 84.3 ± 11.1 years	Pleural effusion+: 49.1% men, 50.9% women; pleural effusion-: 34.7% men, 65.3% women; healthy-control datasets: 27.3% men, 72.7% women; discharge without pleural effusion: 46.7% men, 53.3% women	Multi-frequency TTH BIA	Non-invasive, non-wearable	[12]
5734	Mean with standard deviation	68 ± 14 years	71% men, 29% women	ITH	Implanted	[13]
591	NS	NS	NS	ITH	Implanted	[14]
42	Mean with standard deviation	65.5 ± 9 years	61.9% men, 38.1% women	ITH	Implanted	[15]
39	NS	NS	NS	TH impedance (external and internal)	Implanted	[27]
19	Median with standard deviation	Median: 53 years; SD: 10.6 years	NS	Multi-frequency TH BI	Wearable	[28]
17,886	Mean with standard deviation overall and by condition	Overall: 67 ± 12 years; no HF event: 67 ± 12 years; any HF event: 68 ± 11 years; HF event without readmission: 69 ± 11 years; HF event with readmission: 66 ± 12 years	Overall: 72% men, 28% women; no HF event: 72% men, 28% women; any HF event: 73% men, 27% women; HF event without readmission: 73% men, 27% women; HF event with readmission: 71% men, 29% women	ITH	Implanted	[29]

BI: bioimpedance; BIA: bioimpedance analysis; NS: not specified; HF: heart failure; TH: thoracic; TTH: transthoracic; ITH: intrathoracic.

**Table 3:** Comparative summary of AI techniques and applications in the included studies.

Study Aim	AI Task and Model	Performance Metrics	Validation Method	Ref.
Assess predictors of cardiovascular mortality or HF rehospitalization in ADHF	Outcome prediction/feature-importance analysis (radial-basis and multilayer perceptron neural networks)	Prediction importance reported; no conventional model-performance metric specified	NS	[7]
Detect lung fluid accumulation using transthoracic bioimpedance and heart-rate variability	Binary and multiclass classification (SVM, KNN, DT)	Best binary classification accuracy: 92%	Leave-One-Subject-Out CV	[10]
Predict worsening ADHF using ECG features learned jointly with transthoracic bioimpedance	Regression and binary classification (MLP, SVM; DenseNet121/VGG19-based transfer learning)	Accuracy, precision, recall, F1-score, AUC-PR	5-fold CV; repeated model evaluation	[11]
Estimate the presence of pleural effusion in HF patients	Binary classification (LightGBM)	AUC 0.905; accuracy 0.830; sensitivity 0.755; specificity 0.876	4-fold CV with subject-level separation	[12]
Predict 60-day HF readmission using longitudinal CIED diagnostics	Binary risk classification (1D-CNN and LSTM)	CNN: accuracy 92.8%, sensitivity 76.9%, specificity 97.8%, AUC 0.89; LSTM: accuracy 90.9%, sensitivity 78.6%, specificity 94.7%, AUC 0.88	Independent held-out test set with no patient overlap; stratified 10-fold CV for hyperparameter optimization	[13]
Predict 30-day risk of HF hospitalization using implantable-device diagnostics	Risk prediction/probabilistic risk stratification (BBN)	Sensitivity, specificity, ROC, relative risk, hazard ratio	Internal evaluation using the same source data; independent validation not performed	[14]
Identify physiological feature combinations predictive of HF decompensation	Binary classification and feature selection (GA + FD classifier)	Gmean 88.32%; sensitivity 92.86%; specificity 84%	Leave-One-Out validation	[27]
Estimate thoracic fluid percentage from wearable multifrequency bioimpedance	Regression (GNN with graph-attention mechanisms)	Test MSE 0.8675; R^2^ 0.91; MAE 0.6238	Train/validation/test evaluation; further external validation not reported	[28]

ADHF: acute decompensated heart failure; AI: artificial intelligence; AUC: area under the curve; AUC-PR: area under the precision–recall curve; BBN: Bayesian belief network; CIED: cardiac implantable electronic device; CNN: convolutional neural network; CV: cross-validation; DT: decision tree; FD: Fisher discriminant; GA: genetic algorithm; GNN: graph neural network; KNN: k-nearest neighbors; LightGBM: light gradient boosting machine; LSTM: long short-term memory; MAE: mean absolute error; MLP: multilayer perceptron; MSE: mean squared error; NS: not specified; ROC: receiver operating characteristic; SVM: support vector machine.

**Table 4:** CDSS characteristics presented in the included studies.

Purpose	EHR Integration	Intervention Type	Data Sources	Automation Level	Target Users	Ref.
Estimate pleural effusion and provide information on intrathoracic condition	Independent	Predictive analytics; diagnostic support; web-based information display	Multifrequency transthoracic bioimpedance, clinical variables, laboratory variables	Level 1	NS	[12]
Risk stratification for HF hospitalization and clinician triage	Independent	Predictive analytics; probabilistic risk score; patient-specific decision support	Intrathoracic impedance, AF burden, ventricular rate during AF, nighttime HR, HRV, activity	Level 1	Clinicians	[14]
Support treatment decisions following intrathoracic impedance alerts	Independent	Alerts/reminders; therapeutic decision support; action proposals	Intrathoracic impedance, clinical evaluation, BNP	Level 2	CRT-clinic clinical staff/physicians	[15]
Risk stratification and assessment of temporal changes associated with HF events and recovery	Not specified	Risk assessment; predictive analytics; patient-specific risk information	Intrathoracic impedance, nighttime HR, activity, HRV, AF burden, ventricular rate during AF, CRT pacing, VT episodes and shocks	Level 1	NS	[29]

AF: atrial fibrillation; BNP: B-type natriuretic peptide; CDSS: clinical decision support system; CRT: cardiac resynchronization therapy; EHR: electronic health record; HF: heart failure; HR: heart rate; HRV: heart rate variability; NS: not specified; VT: ventricular tachycardia.

**Table 5:** CFIR-based descriptive mapping of potential implementation considerations across the identified CDSS and decision-support components.

CFIR Domain	Potential Barrier/Implementation Challenge	Potential Facilitator	Ref.
Intervention Characteristics	Limited EHR integration was identified across the included decision-support systems	Independent systems may permit implementation without requiring complete EHR integration	[12,14,15,29]
Intervention Characteristics	Predominantly low automation levels; three of four systems provided information or risk estimates without automated clinical action	Maintenance of clinician oversight may facilitate clinical control over subsequent management decisions	[12,14,29]
Intervention Characteristics	Dependence on heterogeneous and device-specific physiological measurements	Objective BI-derived and multimodal physiological measurements provide patient-specific information on fluid status and HF risk	[12,14,15,29]
Inner Setting	Integration into routine clinical workflow was incompletely evaluated in most studies	Knackstedt et al. specifically evaluated incorporation of an impedance-triggered treatment algorithm into routine CRT follow-up	[12,14,15,29]
Characteristics of Individuals	Intended end-users were not clearly specified in some systems	Clinician-oriented risk information may support prioritization and patient-specific management	[12,14,29]
Process	Formal implementation strategies and prospective implementation-effectiveness evaluations were generally not reported	Existing remote-monitoring and web/cloud-based infrastructures provide potential pathways for future clinical implementation	[12,14,29]

BI: bioimpedance; CDSS: clinical decision support system; CFIR: Consolidated Framework for Implementation Research; EHR: electronic health record.

## Data Availability

No new data were created or analyzed in this study. Data sharing is not applicable to this article.

## Supplementary Materials

The following supporting information can be downloaded at: https://www.mdpi.com/article/10.3390/bioengineering13101227/s1.
