# Supplementary material for: Use of Bioimpedance Monitoring and Artificial Intelligence in Heart Failure: A Scoping Review
Source: Bioengineering (Basel). 2026 Oct 20;13(10):1227. doi: 10.3390/bioengineering13101227 (PMC13701678; doi:10.3390/bioengineering13101227)
Supplement: Supplementary file 1 [file bioengineering-13-01227-s001.zip › bioengineering-4609121-supplementary.pdf]

Data Extraction

Study characteristics were first extracted, including the continent and country in which each study was conducted and the study period, when reported. To enable analysis across both clinical and technical domains, the remaining data items were organized into predefined categories. Clinical data included items related to population characteristics, monitoring and clinical assessment, outcomes and impact, and data collection. Technical data included items related to the methodological and technological characteristics of the systems and approaches evaluated.

To promote consistency during the data extraction process, a standardized extraction form was developed in a shared Excel spreadsheet. The form consisted of predefined sections and fields derived from relevant literature and the research team's expertise. All three reviewers jointly reviewed the form and calibrated their interpretation of each field before full data extraction.

A pilot extraction was then conducted using two randomly selected articles. Each reviewer independently completed the extraction for these studies, and the resulting entries were compared. During this pilot phase, minor adjustments were made to improve the clarity and consistency of the extraction form, including:

- Clarifying field definitions whose meaning was initially ambiguous;
- Adding predefined response options to standardize categorical entries;
- Separating items that combined multiple concepts; and
- Reordering certain fields to improve the extraction workflow.

After these refinements, agreement was reached among the reviewers on the interpretation and application of the extraction fields. Each included study was subsequently extracted independently by two reviewers using rotating reviewer pairs. Extracted data were compared item by item, and discrepancies were resolved through discussion. When consensus could not be reached, a third reviewer adjudicated.

Below, the predefined data items are detailed.

Study characteristics items

- Continent(s) and country(ies) where the study was conducted.
- Years in which the study was conducted. The purpose of this factor is to identify the year or range of years in which the research was taking place.

For studies that did not report this information, the corresponding variables were labeled as ``Not specified''.

Clinical items: HF-related volume status

The following items related to the clinical focus area of HF-related volume status were charted:

- Sample size: number of patients included in each study, or number of measurements/observations when no patient cohort was involved. “Not specified” was used when no sample size was reported.
- Age of patients: recorded as mean, standard deviation, or range, including subgroup-specific values when applicable. Studies without patients were marked “Not applicable,” and those lacking age information were labeled “Not specified.”
- Sex of patients: sex distribution (absolute and/or percentage) was recorded when available. Studies without patient cohorts were marked “Not applicable,” and those not reporting this variable as “Not specified.”
- Main condition studied: main HF-related condition or volume-status manifestation addressed in each article. Studies not centered on a specific condition were marked “Not applicable.”
- Use of biomarkers: whether biomarkers were used for disease detection or monitoring. Absence of information was labeled “Not specified,” and non-relevance as “Not applicable.”
- Clinical outcomes: patient-level outcomes and indicators of improvement or deterioration. If unreported, the field was marked “Not specified,” and if irrelevant to the study aim, “Not applicable.”
- Adverse outcomes targeted for prevention: adverse events such as hospitalization, mortality, or morbidity, when applicable. Missing information was marked “Not specified,” and non-relevance as “Not applicable.”

CDSS and AI characteristics

This category includes information related to the AI techniques used in the selected articles and, when applicable, the characteristics of the CDSS. For studies without a CDSS, all items were marked as “Not applicable.” Items not reported in the articles were labeled “Not specified.”

CDSS Characteristics

- Objective of the CDSS: primary purpose of the system (e.g., diagnosis, treatment, screening, workflow support, medication management, risk assessment).
- Electronic Health Record (EHR) integration: whether integration with an EHR was reported or whether the CDSS was described as operating independently.
- Intervention type: modality through which the CDSS supports decision-making (knowledge-based, alerts/reminders, diagnostic or therapeutic support, medication dosing, image/signal interpretation, workflow support, population health, patient-specific functionalities, interactive systems, predictive analytics, or guideline-based approaches).
- Data sources: types of information used by the CDSS, such as clinical history, laboratory results, images, and monitoring device data.
- Automation level: degree of automation according to the six-level framework in Table 1 ranging from systems that only provide suggestions to fully autonomous systems.
- CDSS user: intended end-users, indicating who is expected to operate or interact with the system.

Table 1: PROPOSED DESCRIPTION OF CDSS AUTOMATION LEVELS, ELABORATED BASED ON [22][23][24][25][26]

Level	Decision	Description

Level 0	No automation	No automated support is provided.
Level 1	Suggestions	Provides diagnostic or treatment suggestions; final decision is made by the clinician.
Level 2	Alerts/reminders	Automatically issues alerts or reminders; requires clinician action.
Level 3	Action proposals	Proposes specific actions that must be reviewed and confirmed by the clinician.
Level 4	Semi-autonomous	Executes actions automatically, while allowing clinician oversight and intervention.
Level 5	Fully autonomous	Executes actions independently without human intervention.

To improve interpretability and reduce ambiguity regarding the proposed CDSS automation levels, the proposed automation-level classification was conceptually grounded in established human automation interaction models [22][23]. The six levels used in this review represent a simplified adaptation for clinical decision-support applications, ranging from no automation to fully autonomous action.

AI techniques

For studies without AI components, all items were marked as “Not applicable.” Items not reported were labeled “Not specified.”

- AI technique: type of method used.
- AI task: specific task addressed by the AI method.
- AI model: model(s) implemented for the corresponding technique or task.
- Performance metrics: metrics used to evaluate model performance.
- Validation strategy: method used to validate or evaluate the AI model.

BI techniques, Characteristics and Monitoring

- Remote monitoring device: device used for remote BI measurements, when reported. Labeled “Not applicable” if no device was used and “Not specified” if not reported.
- Non-remote monitoring device: device used for on-site BI measurements. Marked as “Not applicable” when no measurement device was relevant and “Not specified” when the study did not report the information.
- Monitoring system: type of monitoring (continuous, intermittent, alert-based). Marked “Not specified” when not reported or “Not applicable” when not relevant.
- BI technique: specific BI modality used in the study.
- Electrodes and frequencies: number and placement of electrodes and the measurement frequencies employed. Marked “Not specified” when absent and “Not applicable” when not relevant.

- Sensing platform: the type of system used to acquire BI. Classified as implanted, when impedance was measured by an implanted cardiac device; wearable, when BI was acquired with a body-worn system; and non-invasive non-wearable, when surface electrodes were used without a body-worn system.

BI terminology taxonomy

To ensure consistency and facilitate interpretation across readers from different disciplinary backgrounds, a simplified taxonomy of BI-related terminology was adopted in this review. Six categories were defined based on the measurement configuration, frequency spectrum, and clinical context in which the impedance signal is used:

- Bioelectrical Impedance Analysis (BIA): Conventional whole-body or segmental impedance measurement, typically using hand-to-foot or foot-to-foot electrodes. Commonly used to estimate body composition or fluid status
- Thoracic BI: Non-invasive impedance measured across the thorax with surface electrodes to monitor thoracic fluid content or respiratory dynamics.
- Intrathoracic impedance: Impedance measured by implanted cardiac devices (Implantable Cardioverter-Defibrillator (ICD)/Cardiac Resynchronization Therapy--Defibrillator (CRT-D)) through intracardiac leads and used as a device-derived indicator associated with changes in intrathoracic fluid status.
- BI Spectroscopy (BIS): Multi-frequency impedance measurement over a wide frequency range, enabling estimation of extracellular and intracellular fluid compartments and improved tissue characterization.
- Unspecified electrical BI: Studies reporting "electrical BI" without details on electrode configuration, frequency domain, or measurement modality.

Study design and patient participation

In addition to BI-related variables, the following study-level items were extracted:

- Study design type: classified as prospective, retrospective, monitoring-based, or a combination of these. When the design could not be determined from the article, it was recorded as "Not specified."
- Active patient participation: whether the study required explicit patient involvement in data collection. Recorded as "Yes" when participation was described, "No" when explicitly absent, and otherwise as "Not specified."

Study limitations

Study limitations explicitly reported by the authors were extracted when available. When limitations were not reported, this item was recorded as "Not specified."
